# Antenatally detected fetus in fetu case report

**DOI:** 10.1259/bjrcr.20230001

**Published:** 2023-05-03

**Authors:** Rucha Ulhas Puranik, Priscilla Joshi, Vandana Jahanvi, Abhilasha Tej Handu, Karthik Reddy Puli

**Affiliations:** 1 Department of Radiodiagnosis, Bharati Vidyapeeth Medical College Hospital, Pune, Maharashtra, India; 2 Department of Pediatric Surgery, Bharati Vidyapeeth Medical College Hospital, Pune, Maharashtra, India

## Abstract

Fetus in fetu (FIF) is an extremely rare pathology in which a malformed fetus is located in the body of its twin. It may occur as a result of an aberration of the twinning process. It is important to distinguish this condition from a teratoma. This article emphasizes the importance of the various modalities in the antenatal diagnosis and post-natal follow-up of FIF. An appropriate early intervention if instituted results in a good prognosis. Only few cases of FIF have been reported in medical literature. We present a case which was antenatally diagnosed and proven on histopathology post-natally. This case report illustrates the importance of multimodality imaging techniques in the diagnosis of this condition.

## Introduction

Fetus-in-fetu (FIF) is a rare entity wherein a non-viable fetus becomes enclosed within a normally developing fetus. This has an incidence of 1 per 500,000^1^ births with a 2:1 male predominance. Only 200 cases have been reported so far according to medical literature.^2^ Hence, it is important to diagnose the condition early, preferably antenatally as the prognosis is good if operated early.

## Case report—Imaging findings

A male term baby born at 40 weeks of gestation was sent to Department of Radiodiagnosis, Bharati Vidyapeeth Medical College and Hospital,Pune, India for an ultrasound examination in view of an antenatally detected abdominal mass. He had been delivered via Cesarean section due to meconium stained liquor *in utero*.

**Figure 1.**
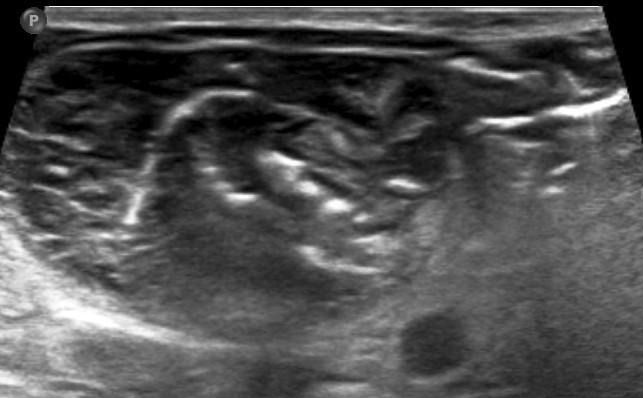
Post-natal USG image showing multiple hyperechoic areas clustered in the center of the mass showing some distal acoustic shadowing.

On abdominal ultrasound, (Figure 1) a hypoechoic mass was seen in the subhepatic region measuring 4.3 × 3.3 x 4.3 cm in the right upper quadrant, anterior to the right kidney. Multiple linear short and long hyperechoic areas were clustered in the center of the mass showing some distal acoustic shadowing. No significant vascularity was seen within this mass which was seen displacing the bowel loops.

**Figure 2.**
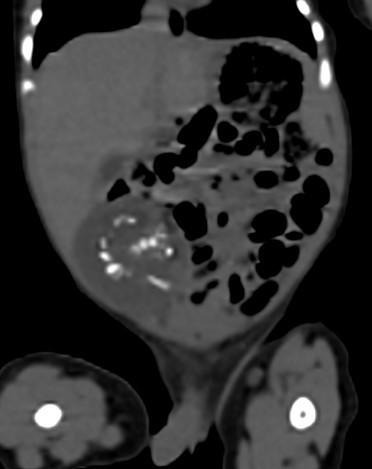
Post-natal CT image showing multiple curvilinear areas bone density structures within the hypodense mass which appear like vertebrae long bones and part of the calvarium.

CT of the abdomen pelvis (Figure 2) confirmed the findings seen on ultrasound of a mass in the subhepatic, right lumbar and right iliac regions which was predominantly cystic with non-enhancing walls (HU value in the range of 15–20). Multiple curvilinear bone-density structures within this lesion resembled the vertebrae, long bones and part of the calvarium. The small bowel loops were displaced towards the left side.

**Figure 3.**
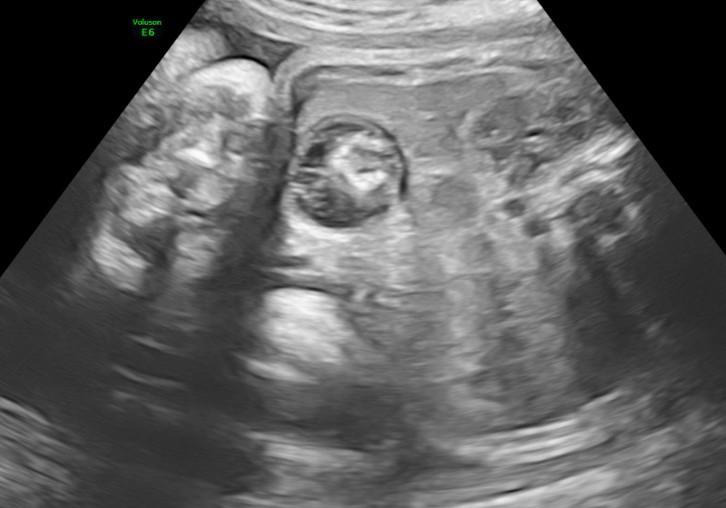
Antenatal USG image showing a well-defined heteroechoic lesion in the right subhepatic region of the fetus.

The baby’s mother had undergone antenatal ultrasound (Figure 3) at Bharati Vidyapeeth Medical College and Hospital, Pune, India at 34 weeks of gestation, which revealed a well-defined heteroechoic lesion in the fetal right hypochondrium. Her first trimester screening showed low risk and anomaly scan done in second trimester outside was reported as normal.

**Figure 4.**
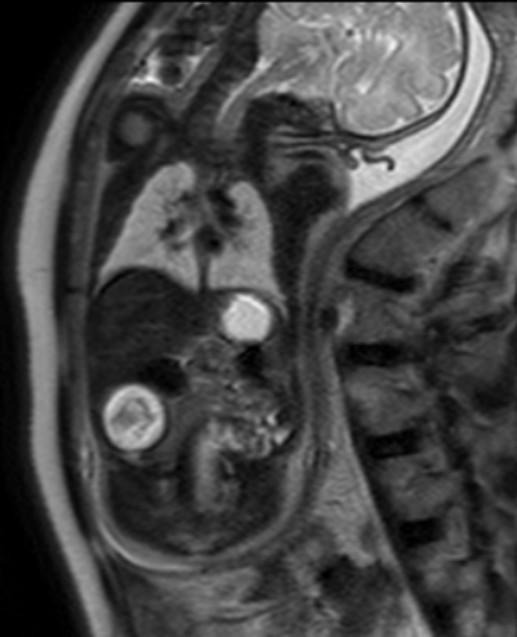
Fetal MRI *T*_2_WI showing a well-defined cystic subhepatic lesion appearing hyperintense with hypointense contents within.

Subsequently, a fetal MRI done (Figure 4) at 35 weeks of gestation showed a lesion which was hyperintense on T2 with hypointense contents within. There were few foci of blooming seen on the GRE images. Possible differential diagnosis were given as FIF, teratoma or a mesenteric cyst with hemorrhagic contents.

Serum *β*-HCG levels of baby was normal.

**Figure 5.**
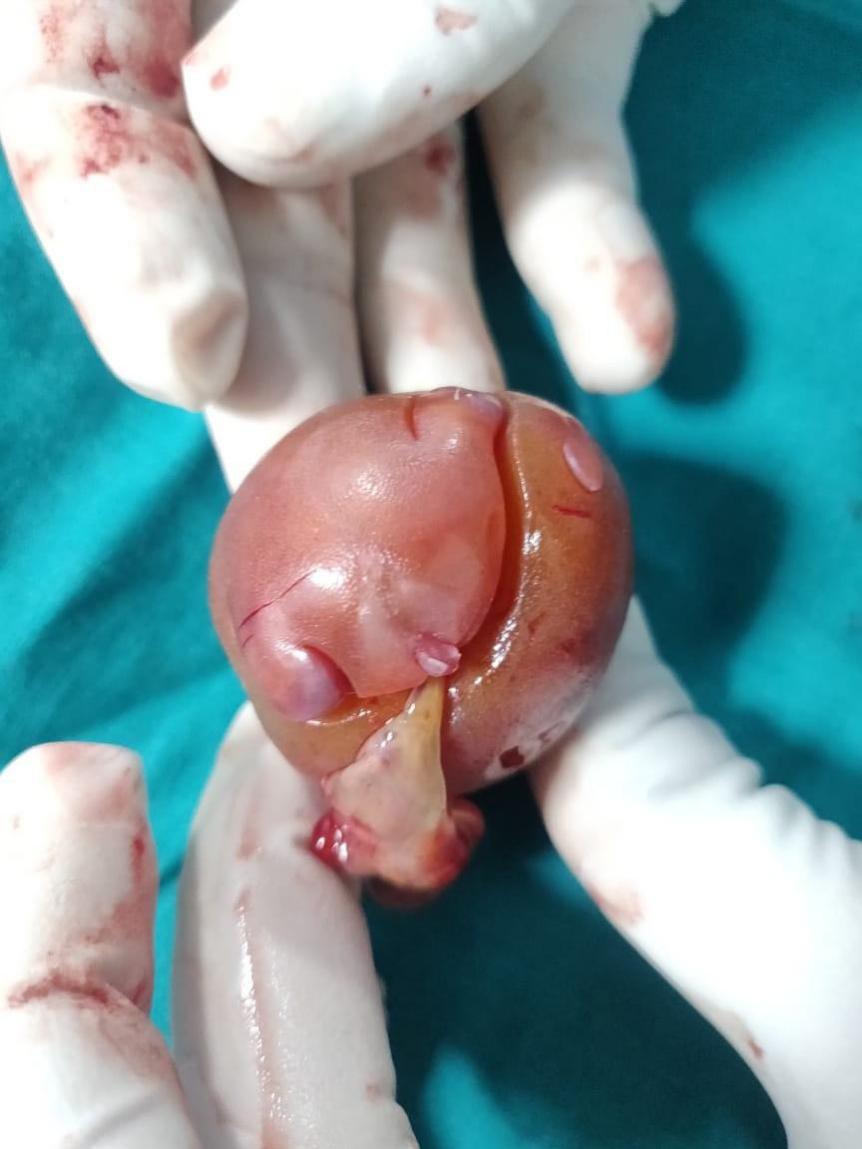
Post-operative image showing a well-defined mass with removed sac and vascular pedicle.

**Figure 6.**
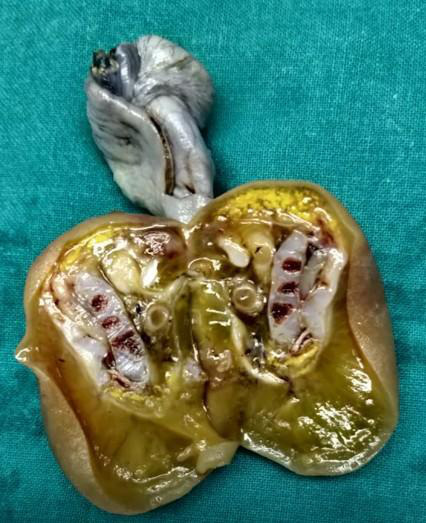
Cut section of resected mass showing vertebral remnants with intestinal loops.

The child was operated on seventh day of life. An oval mass with a sac covering (Figure 5) it was seen with its supply from the right internal iliac artery. On resection, the cut section (Figure 6) showed structures resembling vertebral bodies and intestinal loops.

## Discussion/Summary

The etiology of FIF is controversial but two theories have been proposed. The first is “parasitic twin theory” in which a parasitic fetus is formed inside the body of its host twin and they both share a common blood supply. The parasitic twin is usually malformed and usually dies before birth.

The second theory is of highly differentiated form of teratoma. An aberration in the process of diamniotic, monochorionic, monozygotic twinning may result in unequal division of the totipotent inner cell mass of the developing blastocyst. As a result, a smaller cell mass may remain within a maturing sister embryo.

It can go undiagnosed. Some of the symptoms which are usually reported are abdominal distension, palpable mass, vomiting, poor feeding, jaundice, and/or dyspnea.^3^

Antenatal diagnosis can be done by ultrasound, CT, and MRI.

The presence of the axial bones (cranium, spine, sternum, ribs) in the lesion are a key to the diagnosis of FIF. Gonzalez-Crussi defined FIF as, “any structure in which the fetal form is in a very high development of organogenesis” and linked it to the presence of a vertebral axis^4^ In contrast, teratomas are neoplasms having progressive independent growth, extension and metastasis. There are no signs of presence of a vertebrate axis, germ-layers or organ. It is usually seen as expansive and exophytic mass, heterogeneous in type (solid and cystic), with irregular contours. There is no objective evidence which states that teratomas, no matter how faithfully they reproduce complex structures, be identified with “rudimentary human being”.^5^ Highly organized parts which may occur in a teratoma, *e.g.* teeth, a loop of intestine, a piece of cerebellum, or digits do not pre-suppose a fetus.^6^

Ultrasound is safe and easily available modality for pre-natal examination as there is no risk of radiation. It can also be used for the diagnosis of pre-natal and neonatal FIF. Since FIF and teratoma have similar sonographic features, it may be difficult to differentiate the two and thus this condition can be misdiagnosed. A mature teratoma has ability to grow independently with a high malignant potential.^7^ In recent years, MRI has also been used to diagnose FIF. It is an ideal imaging modality as it can easily identify the soft tissues and organs surrounding the FIF, thereby providing valuable imaging data. As there is no need for contrast and no risk of ionizing radiation, it is considered safe in pregnancy. MR imaging allows better evaluation of intrapelvic extension of the mass and helps in evaluating its contents. It also aids in diagnosis, pre-natal counselling, planning delivery, and perinatal surgical procedures.^8^

Since FIF is generally considered as benign disorder, the recommended treatment is surgical resection of mass.

There have been isolated cases of malignancy/immature contents within the mass which may pose a high risk for the baby. Hence, some surgeons recommend complete resection on a more urgent basis followed by post-operative surveillance of tumor markers for 2 years. If the FIF is not removed, subsequent development of a teratoma may be expected. Hopkins et al had reported a 5-day old boy who had retroperitoneal FIF and later developed a right abdominal mass which proved to be a teratoma with malignant components requiring chemotherapy.^9^ Tumor markers of AFP and *β*-HCG can be used as the basis for patient diagnosis, follow-up observation, and determination of malignant recurrence of FIF.^10^

Histopathological evaluation of the resected mass was performed in our case which showed primitive fetal structures corresponding to the axial skeleton with mature cartilage and other mesenchymal structures.

Informed consent was given by parents.

## Learning points

- In a fetus, the differential diagnosis of an abdominal mass with hyperechoic contents includes two close differentials, *i.e* mature teratoma and FIF. In case of teratoma, only a part of fetus like teeth, a loop of intestine, single long bones or part of limbs are present. The presence of calvarium, long bones and vertebral axis indicates presence of a fetus which points towards FIF.The other differential diagnosis though less likely could be an ovarian cyst with torsion or a hemorrhagic ovarian cyst which in this case was ruled out as the fetus was male.
- Abdominal masses can usually be asymptomatic but at times may cause compression of surrounding structures leading to intestinal obstruction. Hence, timely diagnosis of a FIF will help in ensuring complete resection of the mass thus preventing further complications and reducing the risk of malignancy in baby.
- Our case highlights importance of using a multimodality approach in the antenatal diagnosis and post-natal follow-up in a case of FIF.
